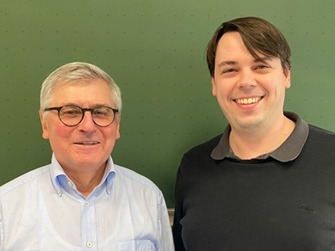# The Huisgen Reaction: Milestones of the 1,3‐Dipolar Cycloaddition

**DOI:** 10.1002/anie.202003115

**Published:** 2020-05-25

**Authors:** Martin Breugst, Hans‐Ulrich Reissig

**Affiliations:** ^1^ Department für Chemie Universität zu Köln Greinstrasse 4 50939 Köln Germany; ^2^ Institut für Chemie und Biochemie Freie Universität Berlin Takustrasse 3 14195 Berlin Germany

**Keywords:** 1,3-dipolar cycloadditions, click chemistry, computational chemistry, heterocycles, reaction mechanisms

## Abstract

The concept of 1,3‐dipolar cycloadditions was presented by Rolf Huisgen 60 years ago. Previously unknown reactive intermediates, for example azomethine ylides, were introduced to organic chemistry and the (3+2) cycloadditions of 1,3‐dipoles to multiple‐bond systems (Huisgen reaction) developed into one of the most versatile synthetic methods in heterocyclic chemistry. In this Review, we present the history of this research area, highlight important older reports, and describe the evolution and further development of the concept. The most important mechanistic and synthetic results are discussed. Quantum‐mechanical calculations support the concerted mechanism always favored by R. Huisgen; however, in extreme cases intermediates may be involved. The impact of 1,3‐dipolar cycloadditions on the click chemistry concept of K. B. Sharpless will also be discussed.

## Graphical abstract

**(3+2) is more than 5**! This Minireview presents milestones of the Huisgen reaction, also known as the 1,3‐dipolar cycloaddition. The historical development, mechanistic aspects, and computational investigations as well as applications in the synthesis of heterocycles, natural products, and biologically active compounds, biosynthesis, and in click chemistry are discussed.

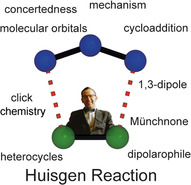

*“The elucidation of a reaction mechanism does not provide conclusions for eternity, rather it is a process in steps intending an increasingly deeper understanding of the reaction processes.” –* Rolf Huisgen (translation by the authors), 1960.1a

## 1. Introduction

**Scheme 1:**
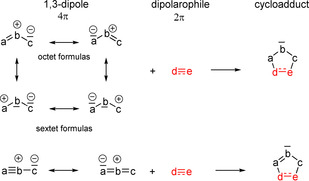
(3+2) Cycloadditions of 1,3‐dipoles “abc” to dipolarophiles “de” to give five‐membered heterocycles (only lone pairs relevant for the 4π system are drawn).

In several lectures in 1960 Rolf Huisgen introduced the concept of 1,3‐dipolar cycloadditions providing five‐membered heterocycles; the first reports were published shortly afterwards.^1^ In analogy to the Diels–Alder reaction, a 1,3‐dipole reacts as a 4π system with the general formula “abc” with a dipolarophile “d=e” or “d≡e” (delivering 2π electrons) in a (3+2) cycloaddition to give the five‐membered product (Scheme 1). The 1,3‐dipoles “abc” cannot be described by neutral octet formulas, but they bear a positive charge at the center atom “b”; the two sextet formulas with neutral “b” explain the choice of the name 1,3‐dipole for these compounds.^2^ The four formulas depicted in Scheme 1 reflect the ambivalent character of 1,3‐dipoles with nucleophilic and electrophilic properties; possible diradical mesomeric formulas are not shown in this illustration. These heteroallyl or heteropropargyl anion systems react with a variety of double‐ and triple‐bond systems to provide five‐membered cycloadducts with neutralization of the formal charges.

**Scheme 2:**
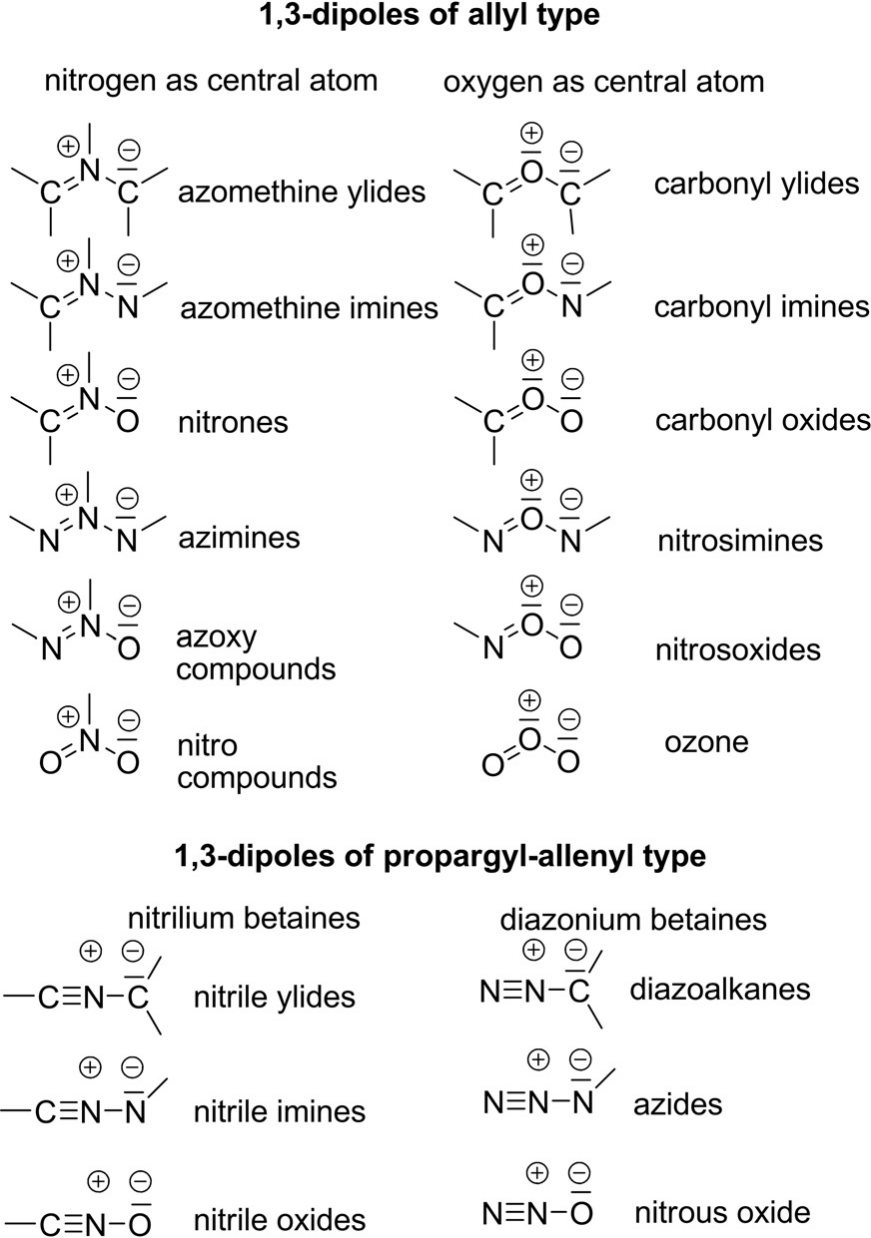
Classification of 1,3‐dipoles (the Lewis structures show only lone pairs relevant for the 4π system).

R. Huisgen and his co‐workers recognized that 1,3‐dipoles “abc” can be subdivided in two comprehensive classes (Scheme 2). For the twelve allyl type 1,3‐dipoles, the central atom “b” can be nitrogen or oxygen. For the six allenyl‐propargyl type 1,3‐dipoles, only nitrogen is possible; their second orthogonally located double bond causes the linear structure, but it is not involved in the cycloaddition. This system was used not only to classify already known compounds but it also helped identify gaps and led to the development of new routes to hitherto unknown 1,3‐dipoles. In Scheme 2 only elements of the second period of the periodic table are considered. The involvement of sulfur, phosphorus, or other hetero atoms broadens the scope of possible 1,3‐dipoles.^3^

At the start of the studies in the Munich laboratories (1957–1959), only nine of the eighteen 1,3‐dipoles were known as compound classes and for only five of these had cycloadditions been reported (diazoalkanes, azides, nitrones, nitrile oxides, ozones). The recognition of common reaction features and their systematic expansion made the Huisgen reaction^4^ one of the most important principles for the synthesis of heterocyclic compounds. The reaction was also employed for the preparation of natural products, and it was involved in new, unexpected applications in very different fields of science through the development of click chemistry.

Voluminous compilations, edited by A. Padwa, describe in ca. 2500 pages with more than 7500 citations the results up to the early 1980s and 2000.^5^ The evolution of the research area is presented in the insightful introductory chapter by R. Huisgen.^6^ He later supplemented many aspects in his autobiography.^7^ In this Review, we discuss selected milestones along the way to the Huisgen reaction and we also comment on the most important synthetic and mechanistic advancements (also see the summarizing Tables 1 and 2).

**Table 1:** Important discoveries and publications concerning 1,3‐dipolar cycloadditions during the first 100 years—from phenyl azide to “Münchnones”.

Year	Discovery/Research subject	Main author	Location	Ref.
1864	Synthesis of phenyl azide (“Diazobenzolimid”)	P. Griess	London/Marburg^[a]^	9
1883	Synthesis of ethyl diazoacetate	T. Curtius	Munich	10
1888	Cycloadditions of methyl diazoacetate to unsaturated carboxylic acid esters	E. Buchner	Erlangen	11
1889	Cycloaddition of methyl diazoacetate to acetylene dicarboxylic diester^[b]^	E. Buchner	Munich	12
1890	Cycloaddition of a nitrone to phenyl isocyanate	E. Beckmann	Leipzig	13
1893	Cycloaddition of phenyl azide to acetylene dicarboxylic diester	A. Michael	Torwood Bonchurch^[c]^	14
1894	Preparation of diazomethane and first cycloadditions	H. von Pechmann	Munich	109
1894	Synthesis of benzonitrile oxide	A. Werner	Zürich	110
since1903	Ozonolyses of alkenes	C. Harries	Berlin	16
1910	Additions of hydrazoic acid to ethyne and hydrogen cyanide	O. Dimroth	Munich	15
1935	Synthesis of mesoionic “Sydnones”	J. C. Earl	Sydney	19
1938	Review: “Systems Capable of Undergoing 1,3‐Additions”	L. I. Smith	Minneapolis	22
1946	Cycloadditions of benzonitrile oxide to alkenes	A. Quilico	Milan	25
1959	Intramolecular nitrone–alkene cycloadditions	N. A. LeBel	Detroit	27
1960	Generation of nitrile oxides from nitroalkanes	T. Mukaiyama	Tokyo	26
1960	Concept of 1,3‐dipolar cycloadditions	R. Huisgen	Munich	1
since 1960	First cycloadditions of previously unknown 1,3‐dipoles: azomethine ylides, azomethine imines, nitrile ylides, and nitrile imines	R. Huisgen	Munich	28, 29, 30, 31
1961	First 1,3‐dipolar cycloadditions to an aryne	R. Huisgen	Munich	32
1963	Review: “1,3‐Dipolar Cycloaddition—Past and Future”	R. Huisgen	Munich	33
1964	Synthesis and cycloadditions of “Münchnones”	R. Huisgen	Munich	34

[a] The experiments were performed either in London (with A. W. Hofmann) or in Marburg (with H. Kolbe); P. Griess did not specify exactly where the individual experiments were done. [b] “*The compounds combine with extreme violence under appearance of fire*; *therefore dilution with ether is required*.” (citation from ref. 12a, translated by the authors). [c] Isle of Man, United Kingdom.

**Table 2:** Important publications concerning mechanistic aspects of the Huisgen reaction.

Year	Research Subject	Main author	Location	Ref.
1932	Stereospecific cycloadditions of diazomethane	K. von Auwers	Marburg	18
1934	Formula of diazomethane with correct Lewis structure	H. Boersch	Vienna	111
1953	Mechanism of ozonolyses of alkenes	R. Criegee	Karlsruhe	17
1954	Cycloadditions of diazomethane and phenyl azide to strained cycloalkenes	K. Ziegler	Mülheim/Ruhr	112
1957–1959	Mechanism of diazoalkane and aryl azide cycloadditions to norbornene derivatives	R. Huisgen	Munich	24
1960	General concept of 1,3‐dipolar cycloadditions	R. Huisgen	Munich	1
1961	Reaction of cyclooctyne with phenyl azide	G. Wittig	Heidelberg	104b
1963	Review: “Kinetics and Mechanism of 1,3‐Dipolar Cycloadditions”	R. Huisgen	Munich	39
1964	First calculations of activation enthalpies of alkene–diazoalkane cycloadditions	O. E. Polansky, P. Schuster	Vienna	52
1965	First publication concerning the conservation of orbital symmetry	R. B. Woodward, R. Hoffmann	Cambridge/USA	40
1967	Stereospecific ring‐opening of aziridines to give azomethine ylides and their cycloadditions	R. Huisgen	Munich	42
1968	Proposal of the diradical mechanism for 1,3‐dipolar cycloadditions and Diels–Alder reactions	R. A. Firestone	Rahway	43
1969	Woodward–Hoffmann Rules	R. B. Woodward, R. Hoffmann	Cambridge/USA, Ithaca	41
1971	Application of the frontier orbital model for the classification of reactivities in 1,3‐dipolar cycloadditions and Diels–Alder reactions	R. Sustmann	Münster	53
1972	Interpretation of the regioselectivity in 1,3‐dipolar cycloadditions with the frontier orbital model	J. Bastide K. N. Houk	Perpignan Baton Rouge	54 55
1975	Ab initio calculations of the reaction mechanism of the 1,3‐dipolar cycloaddition	D. Poppinger	Canberra	57
1978	Record stereospecificity of diazomethane cycloadditions	R. Huisgen	Munich	46
1986	First two‐step 1,3‐dipolar cycloadditions of thiocarbonyl ylides via zwitterions	R. Huisgen	Munich	50
2003	Concluding review: “1,3‐Dipolar Cycloadditions: Concertedness, Yes or No?”	R. Huisgen	Munich	113
2007	Distortion/interaction model for interpretation of 1,3‐dipolar cycloadditions	K. N. Houk	Los Angeles	66
2012	Olefin–carbonyl metathesis with the help of in situ generated azomethine imines	T. H. Lambert	New York	114
2018	Analysis of the concerted reaction mechanism of diazoalkane–alkene cycloadditions employing nucleophilicity–electrophilicity parameters	H. Mayr, A. R. Ofial, H. Zipse	Munich	48
2018	Last publication of R. Huisgen concerning 1,3‐dipolar cycloadditions: regioselectivity of heteroatom‐substituted alkynes with diazoalkanes	M. Breugst, R. Huisgen, H.‐U. Reissig	Cologne, Munich, Berlin	69

## 2. The Beginnings

**Figure 1:**
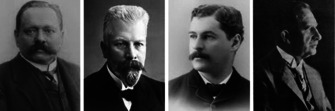
Theodor Curtius, Eduard Buchner, Arthur Michael, and Otto Dimroth.

**Scheme 3:**
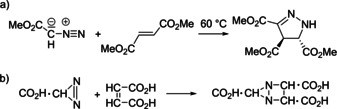
a) The first 1,3‐dipolar cycloaddition of methyl diazoacetate with fumaric acid diester; b) reaction as published by E. Buchner (Erlangen, 1888).

Already in the early 19th century, J. L. Gay‐Lussac and J. von Liebig studied “fulminic acid” H‐CNO (formonitrile oxide), the parent compound of nitrile oxides; however, the propensity of nitriles oxides to undergo cycloadditions was explored only much later.^8^ Historically more important are the classes of azides and diazoalkanes, for which the first (3+2) cycloadditions were reported. In 1864 P. Griess prepared phenyl azide^9^ without investigating its ability to react in cycloadditions. Important early contributions came from the Munich laboratories (at that time “Chemisches Laboratorium der Königlichen Akademie der Wissenschaften zu München”). In 1883 T. Curtius (Figure 1) prepared ethyl diazoacetate, the very first diazoalkane^10^ and he encouraged his colleague and friend E. Buchner to examine its reactions with unsaturated carboxylic acid esters. In 1888 E. Buchner reported the reaction of methyl diazoacetate with dimethyl fumaric acid, the first 1,3‐dipolar cycloaddition (Scheme 3 a).^11^ The equation as provided by E. Buchner is shown in Scheme 3 b, with a cyclic formula for the diazo compound, which should be assessed considering the lack of knowledge about structural chemistry and bonding theory in those days. No better description of diazoalkanes was available. The bicyclic structure of the product contradicted the formation of a cyclopropane derivative after heating, but E. Buchner already recognized the formation of five‐membered heterocycles (pyrazole derivatives) when he continued these studies.^12^

**Scheme 4:**
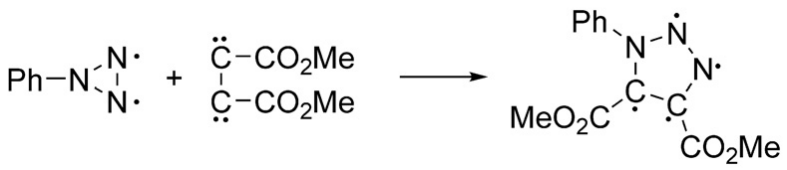
A. Michael′s equation for the first azide–alkyne cycloaddition providing a 1,2,3‐triazole (Torwood Bonchurch, Isle of Man, 1893).

Starting in 1890, E. Beckmann investigated nitrones, for which he also suggested cyclic structures; a 1,3‐dipolar cycloaddition with phenyl isocyanate can be found among the reactions studied.^13^ The formulas used by A. Michael (Figure 1) for the first cycloaddition of an organic azide with an alkyne look strange from today's perspective (Scheme 4);^14^ multiple bonds are represented by dots, the azide is shown as a cyclic compound.

O. Dimroth (Figure 1) still used similar structures in 1910, when he described the additions of hydrazoic acid and phenyl azide to ethyne and hydrogen cyanide, respectively, to afford triazole and tetrazole derivatives.^15^ In about the same period, C. Harries started his systematic studies of the cleavage of multiple bonds with ozone.^16^ The mechanistic interpretation by R. Criegee involving (3+2) cycloadditions and cycloreversions followed only many years later.^17^

**Scheme 5:**
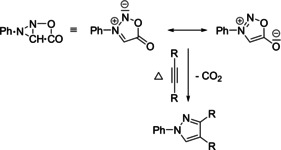
A “Sydnone” as depicted by J. C. Earl and A. W. Mackney (left); actual structure of this mesoionic compound and its reaction with alkynes to afford pyrazoles by cycloaddition/cycloreversion according to R. Huisgen and H. Gotthardt.

The first stereospecific 1,3‐dipolar cycloadditions were reported by K. von Auwers et al., who obtained two different (diastereomeric) cycloadducts when they reacted diazomethane with *cis*/*trans*‐isomeric unsaturated carboxylic acid esters (Table 2).^18^ Remarkable is also the preparation of “Sydnones” from *N*‐nitroso amino acids and acetic anhydride, which were described as bicyclic compounds and not as mesoionic systems bearing an azomethine imine moiety (Scheme 5).^19^ The ability of this novel class of compounds to undergo (3+2) cycloadditions was discovered only after 1960 and proven in numerous publications.^20^, ^21^ The first review summarizing the (3+2) cycloadditions known in 1938 was published by L. I. Smith in *Chemical Reviews*.^22^ However, he did not distinguish between 1,3‐cycloadditions and 1,3‐additions of HX species, and therefore a general reaction principle was not recognizable.^23^

## 3. The Concept, First Applications, the Interpretation

In studies of the cycloadditions of diazoalkanes and aryl azides to strained olefins (norbornene derivatives), the high rate of these reactions attracted attention in the Munich laboratories in 1957–1959. These observations were not in agreement with the previously assumed reaction mechanism via (stabilized) 1,5‐zwitterions.^24^ Thus, a concerted process without involvement of intermediates was suggested. The search for compound classes related to diazoalkanes led to the systematic description of 1,3‐dipoles (see Schemes 1 and 2) and to the outline of the concept of 1,3‐dipolar cycloadditions. Based on this knowledge, many literature results could be classified and explained, e.g., the nitrile oxide cycloadditions of the Italian school around A. Quilico^25^ and those of T. Mukaiyama et al.,^26^ as well as the intramolecular nitrone–alkene cycloadditions reported by N. LeBel.^27^

Most importantly, new 1,3‐dipoles were designed and examined, e.g., azomethine ylides^28^ and azomethine imines,^29^ as well as nitrile ylides^30^ and nitrile imines,^31^ unexplored entities at that time. They were mostly generated by 1,3‐elimination reactions from acyclic precursors or from five‐membered heterocycles. Their (3+2) cycloadditions with alkenes, alkynes, and heteroatom‐containing dipolarophiles provided a broad spectrum of nitrogen heterocycles, in particular pyrrole and pyrazole derivatives. With benzyne, an aryne was used for the first time as a perfectly suitable dipolarophile.^32^ A comprehensive summary of these results was reported in 1963;^33^ this review is still Rolf Huisgen's most cited publication.

**Scheme 6:**
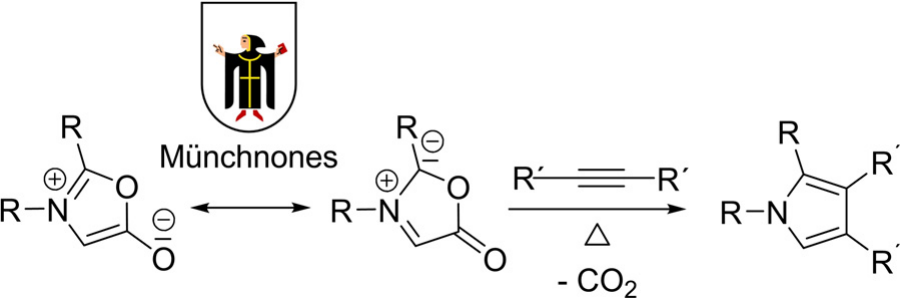
(3+2) Cycloaddition of “Münchnones” to alkynes according to R. Huisgen and H. Gotthardt.

Stimulated by the successful cycloadditions of the mesoionic “Sydnones”,^20^ the “Münchnones” were designed in 1964; they contain an azomethine ylide subunit and opened a novel route to pyrrole derivatives (Scheme 6).^34^, ^35^

In the meantime, azomethine ylides are very frequently employed in regio‐ and stereoselective syntheses of pyrrolidine derivatives and they are so well established that the discoverer and eponym of these reactive intermediates is generally no longer cited.^36^ The photochemical generation of nitrile ylides from 2*H*‐azirines, first reported by A. Padwa^37^ and H. Schmid,^38^ turned out to be a synthetically very fertile route to pyrrole derivatives and other five‐membered heterocycles.

**Figure 2:**
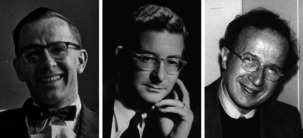
Rolf Huisgen (ca. 1960), Robert B. Woodward (1955), and Roald Hoffmann (1973).

**Scheme 7:**
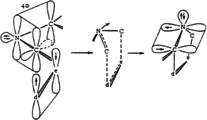
Approach of an azomethine ylide to a dipolarophile during a 1,3‐dipolar cycloaddition (reproduction of the orbital illustration in ref. 39).

Besides the enormous synthetic potential for the preparation of heterocycles, which was rapidly explored in the Munich laboratory, the involved reaction mechanisms were also of central interest. The stereospecificity, the generally low solvent dependency of the reaction rates, and the activation parameters of many reactions were in agreement with a concerted bonding process that was later confirmed by kinetic isotope effects, observed regioselectivities, and reaction rates.^6^, ^7^, ^39^ Already in 1963 an orbital illustration was presented that shows 1,3‐dipole and dipolarophile approaching each other in two parallel planes to achieve a favorable overlap of the π‐systems (Scheme 7). The close relationship with the Diels–Alder reaction was underlined by the theoretical treatment of concerted cycloadditions published in 1965 by R. B. Woodward and R. Hoffmann (Figure 2).^40^ In their reviews, the 1,3‐dipolar cycloaddition and the Diels–Alder reactions were presented side by side as thermally allowed suprafacial–suprafacial [4π+2π] processes.^41^

**Scheme 8:**
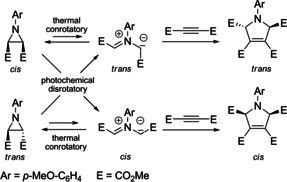
Stereospecific electrocyclic ring‐openings of aziridine derivatives to give azomethine ylides and their (3+2) cycloadditions.

The generation of a range of 1,3‐dipoles can also be achieved by the electrocyclic ring‐opening of three‐membered heterocycles (aziridines, epoxides, 2*H*‐azirines). The investigations performed in Munich dealing with the ring‐opening reactions of aziridine dicarboxylic esters are meanwhile textbook content, since they perfectly illustrate the complementarity of thermal and photochemical reactions and hence the validity of the Woodward–Hoffmann rules for these electrocyclic reactions. Furthermore, they prove the stereospecificity of the subsequent 1,3‐dipolar cycloadditions. The two *cis*/*trans*‐isomeric aziridine dicarboxylic esters undergo a thermal conrotatory ring‐opening to *trans*‐ or *cis*‐configured azomethine ylides, each of which can be stereospecifically trapped by acetylene dicarboxylic ester to furnish stereoisomeric 2,5‐dihydropyrrole derivatives (Scheme 8). By photochemical excitation, the disrotatory ring‐openings generate the configurational isomeric 1,3‐dipoles that provide the complementary diastereomeric cycloadducts in the subsequent thermal step.^42^

**Figure 3:**
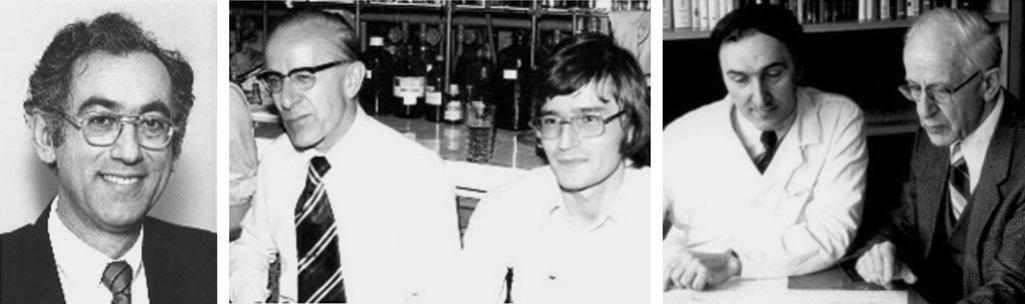
To be concerted, or not to be: that is the question. Raymond A. Firestone (1984), Rolf Huisgen with Hans‐Ulrich Reissig (1975), and with Grzegorz Mlostón (1991).

**Scheme 9:**
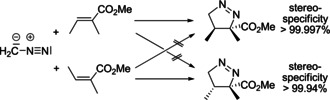
Stereospecific (3+2) cycloadditions of diazomethane to methyl esters of tiglic and angelic acid.

However, the concerted reaction mechanism of the 1,3‐dipolar cycloaddition and the Diels–Alder reaction was also challenged. In 1968 R. A. Firestone (Figure 3) published his first report in which he interpreted literature results of these cycloadditions in favor of a stepwise process with short‐lived 1,5‐diradical intermediates.^43^, ^44^ The long‐lasting and heated discussion between him, R. Huisgen,^45^ and others instigated many additional experimental and theoretical studies that considerably helped clarify the reaction mechanisms. As an example, the cycloaddition of diazomethane with α,β‐unsaturated carboxylic esters was reexamined and the degree of stereospecificity was advanced to a record level (Scheme 9).^46^ In another key publication R. A. Firestone, K. N. Houk et al. demonstrated that the cycloadditions of benzonitrile oxide to specifically deuterated ethenes also proceed with high stereospecificity.^47^ Recent kinetic investigations by H. Mayr, A. R. Ofial, H. Zipse, and colleagues involving aryl diazoalkanes are further impressive evidence for the concerted nature of the Huisgen reaction.^48^, ^49^

**Scheme 10:**
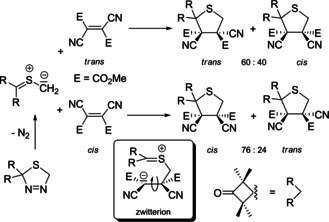
(3+2)‐Cycloadditions of a thiocarbonyl ylide with *cis*/*trans*‐isomeric dipolarophiles: the first two‐step 1,3‐dipolar cycloaddition with a zwitterionic intermediate.

In 1986 the first (3+2) cycloaddition unequivocally involving zwitterionic intermediates was discovered in R. Huisgen's laboratory.^50^ The report published with G. Mloston (Figure 3) and E. Langhals50a describes the reaction of a thiocarbonyl ylide sterically hindered on one side (generated in situ from a thiadiazoline by nitrogen elimination, i.e., by 1,3‐dipolar cycloreversion) with extremely electrophilic dipolarophiles, and a clear violation of the stereospecificity was recorded. Starting with the *trans*‐alkene a 60:40 mixture of the two tetrahydrothiophene derivatives was obtained, whereas the *cis*‐alkene provided a 24:76 ratio of the two products (Scheme 10). This violation of the stereospecificity is plausibly explained by the very well stabilized zwitterion whose ring‐closure to the five‐membered ring is decelerated due to steric hindrance by the substituents R, CO_2_Me, and CN, thus allowing rotation around the C−C single bond. With other very electron‐deficient dipolarophiles, for example, tetracyanoethylene and the 1,2‐bis(trifluoromethyl)ethylene‐1,2‐dicarbonitriles, the intermediate zwitterionic species provide seven‐membered‐ring ketene imines with interesting subsequent reactions.50c, 50d, 51

## 4. Theoretical Investigations

**Figure 4:**
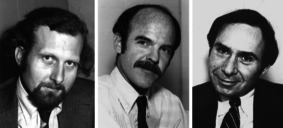
Reiner Sustmann (1974), Kendall N. Houk (1983), and Albert Padwa (1983).

**Scheme 11:**
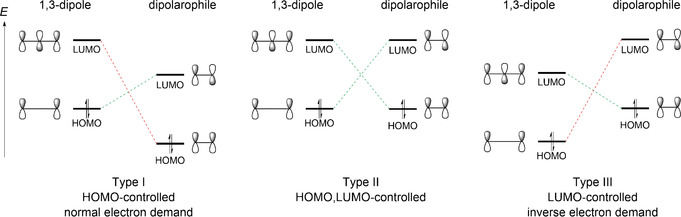
Classification of (3+2) cycloadditions according to the dominant interaction in the frontier molecular orbitals of the 1,3‐dipole. The concept of orbital control refers to the orbital of the 1,3‐dipole.

Consistent with the importance of Huisgen reactions, computational chemists started to investigate this transformation shortly after its discovery. In 1964, O. E. Polansky and P. Schuster amongst others employed Hückel theory to calculate the activation enthalpies of the 1,3‐diploar cycloadditions of 1,1‐diphenyldiazomethane to different alkenes.^52^ In 1971, the group of R. Sustmann (Figure 4)^53^ and shortly thereafter the groups of J. Bastide^54^ and K. N. Houk^55^ relied on frontier molecular orbital theory on the basis of the Klopman–Salem equation to describe (3+2) cycloadditions. These transformations can be classified in three reaction types by the relative energetic position of the HOMO (highest occupied molecular orbital) and the LUMO (lowest unoccupied molecular orbital) (Scheme 11): Type I (HOMO‐controlled, normal electron demand), type II (HOMO,LUMO‐controlled), and type III (LUMO‐controlled, inverse electron demand). With this information, reaction rates and regioselecivities of different cycloadditions could be classified and (partially) explained.

The early sophisticated computational studies on mechanistic aspects of (3+2) cycloadditions were driven by the question of concerted or stepwise pathways. While MNDO calculations by M. J. S. Dewar and colleagues suggested a stepwise reaction between fulminic acid (HCNO) and acetylene with a diradical intermediate,^56^ ab initio calculations by D. Poppinger indicated a concerted pathway.^57^ More accurate multiconfiguration self‐consistent field (MC‐SCF) calculations by M. A. Robb and colleagues strongly supported the concerted nature of this transformation.^58^ Since the comprehensive investigations by M. T. Nguyen et al.^59^ relying on CCSD(T) and CASSCF/CASPT2, Huisgen reactions are generally considered to proceed as concerted reactions.^60^ Exceptions are possible when stabilized zwitterions as described above in Scheme 10 can be formed. For the reaction discussed in Scheme 10, also DFT calculations at the B3LYP/6‐31G(d) level predict a stepwise reaction.^61^

**Scheme 12:**
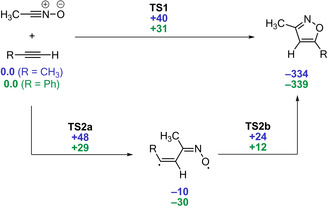
Relative energies of the concerted and stepwise (3+2) cycloaddition of nitrile oxides and alkyl‐substituted (blue) or aryl‐substituted (green) alkynes (CASPT2/def2‐TZVP//B2PLYP‐D2/6‐31G(d) in kJ mol^−1^).^62^

An interesting change in the reaction mechanism was predicted by G. Haberhauer, R. Gleiter, and S. Woitschetzki for (3+2) cycloadditions of nitrile oxides based on CASPT2/def2‐TZVP//B2PLYP‐D2/6‐31G(d) calculations.^62^ The addition to alkyl‐substituted alkynes (Scheme 12) and to alkenes (not shown in Scheme 12) proceeds via the classical, concerted reaction mechanism (blue numbers in Scheme 12). In contrast, transition states for the formation of *anti*‐configured 1,5‐diradicals are similar or even lower in energy compared to the concerted process for aryl‐substituted alkynes (green numbers in Scheme 12). For these stepwise reactions, the calculations predict the addition to be the rate‐limiting step, as the subsequent rotation of the diradical proceeds with a significantly smaller barrier. The authors consider the detection of alkynyl oximes as byproducts in reactions between aryl alkynes and nitrile oxides as additional support for the findings.^51^, ^63^, ^64^ In fact, the corresponding transition state for the hydrogen transfer is located only 20 kJ mol^−1^ above the diradical and could be overcome in this reaction.^62^ However, investigations by two Italian groups^65^ had indicated in the 1970s that no kinetic isotope effect was observed in the reaction of deuterated and non‐deuterated phenyl acetylene, which would be expected for a stepwise reaction.

**Scheme 13:**
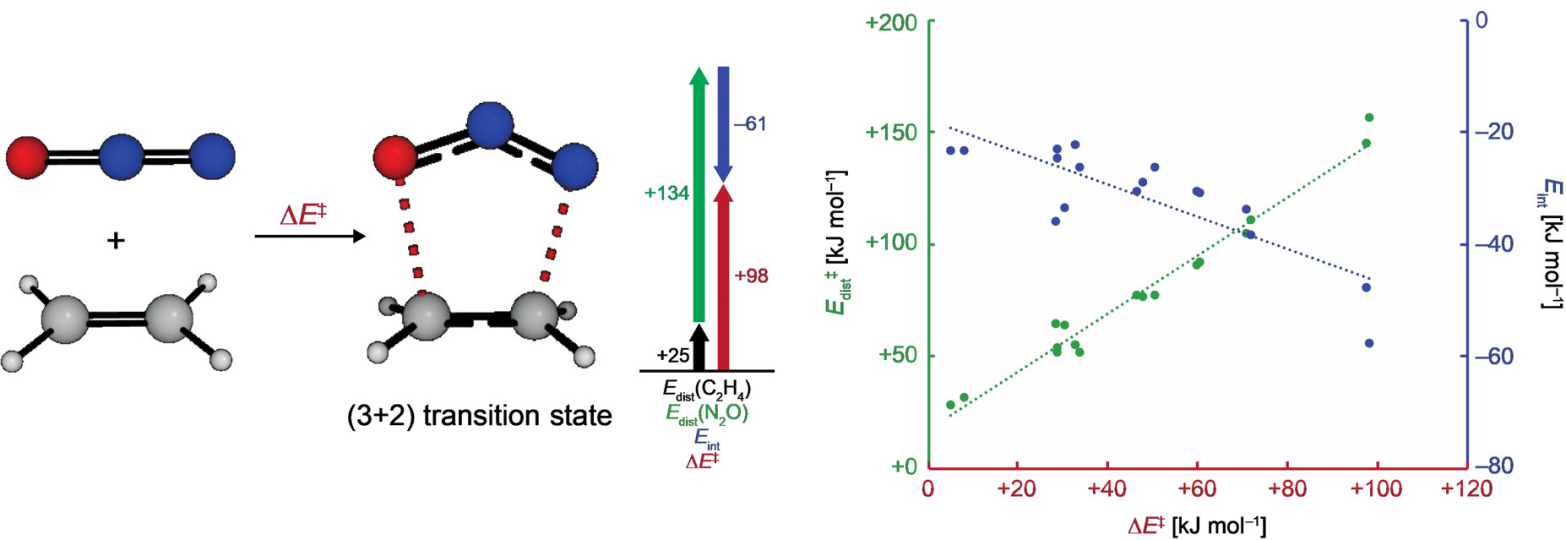
The distortion/interaction model applied to the reaction of ethylene and N_2_O (left) and a correlation of the activation energies (Δ*E* ^≠^) with the distortion energies (Δ*E*_dist_) and interaction energies (Δ*E*_int_) for a series of (3+2) cycloadditions (right). Values are taken from ref. 66b.

A new explanation of the reactivity observed in Huisgen reactions was introduced in 2007/2008 by D. H. Ess and K. N. Houk.^66^ Reactions of nine different 1,3‐dipoles with ethylene and acetylene were analyzed at the CBS‐QB3 level. According to these investigations, the cycloadditions of the different dipoles proceed with either dipolarophile through almost identical activation barriers (e.g., with HN_3_: Δ*H* ^≠^=85 kJ mol^−1^ for ethylene; Δ*H* ^≠^=84 kJ mol^−1^ for acetylene). Based on the relative energies of the frontier molecular orbitals, one would expect a much faster reaction for ethylene as this possesses a energetically higher HOMO (−10.5 eV) and lower LUMO (+1.5 eV) compared to acetylene (−11.5 and +2.5 eV).^67^ To explain this result, the authors applied the distortion/interaction model (also known as the activation‐strain model)^68^ to better understand the reactivities in these reactions. According to this model, the activation energy (Δ*E* ^≠^) is composed of the distortion energy (Δ*E*_dist_) and the interaction energy (Δ*E*_int_) of the reagents (Scheme 13). The distortion energy is the energy required to distort the starting materials into their transition‐state geometries. In their investigations, the authors observed that the calculated activation barriers correlate much better with the distortion energies (*r* ^2^=0.973) rather than with the interaction energies (*r* ^2^=0.679). This led the authors to the conclusion that the distortion energy “*is the major factor controlling the reactivity differences of 1,3‐dipoles*.”^68^ In the last publication of R. Huisgen in 2018, a similar analysis at the DLPNO‐CCSD(T)/aug‐cc‐pVTZ/CPCM//M06‐2X‐D3/6‐311 +G(d,p)/CPCM level also proved useful to explain the regioselectivity of the previously not understood (3+2) cycloadditions of heteroatom‐substituted alkynes with diazoalkanes.^69^

In 2019, T. A. Hamlin, L. Visscher, F. M. Bickelhaupt, and colleagues analyzed (3+2) cycloadditions of 24 nitrogen‐containing 1,3‐dipoles with ethylene.^70^ Initially, all cycloadditions were investigated with different DFT methods with the help of QMflows,^71^ a program for automated workflows of quantum‐mechanical calculations. With respect to the very reliable G3B3 method, smallest errors in activation and reaction energies were found for BP86/TZ2P calculations. Theoretical studies at this level subsequently revealed that the reactivity increases with the number of nitrogen atoms in the 1,3‐dipole (nitrile ylide < nitrile imine < diazomethane < hydrazoic acid). Further investigation with the activation‐strain model as well as with energy decomposition analyses indicate that in these reactions differences in distortion energies cannot be used to explain the different reactivities of 1,3‐dipoles. Instead different interaction energies are responsible for the reactivities in these examples. These interactions are primarily caused by different orbital interactions between the HOMO of the 1,3‐dipole and the LUMO of the dipolarophile. With an increasing number of nitrogen atoms, the energy of the HOMO decreases, which in turn leads to an increase of the HOMO–LUMO gap. From a theoretical perspective, the whole reaction coordinate must be analyzed and not only the transition state. This is particularly important for reactions where the relative position of the transition state shifts along the reaction coordinate.

In summary, more than 50 years of quantum‐mechanical analysis come to the conclusion that Huisgen reactions in general proceed as concerted processes in accordance with experimental results. Stepwise reactions can only be expected when intermediates benefit from exceptional stabilization.

## 5. Applications in Stereoselective Syntheses

**Scheme 14:**
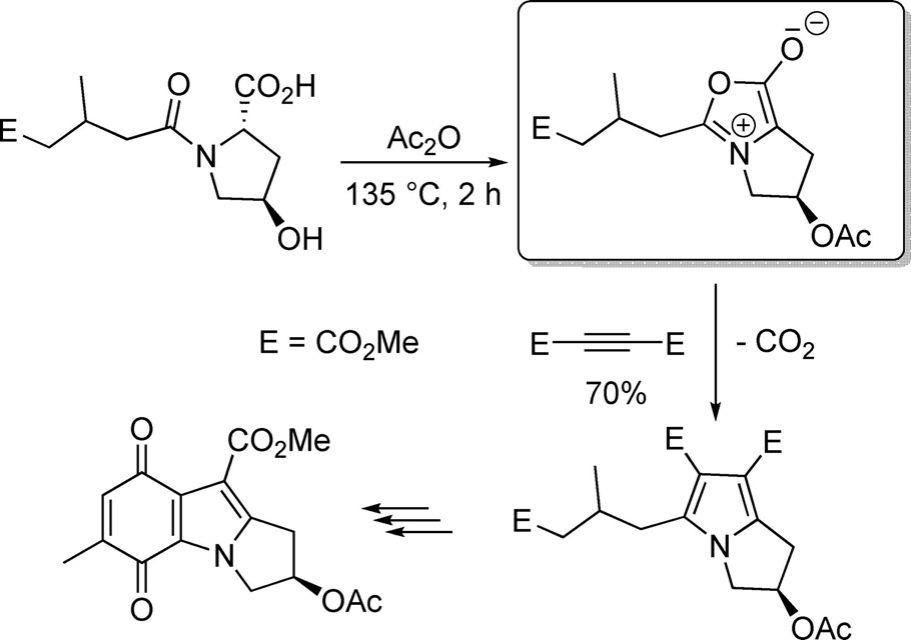
Generation of a “Münchnone” and cycloaddition to provide a pyrrole derivative.

The tremendous potential of Huisgen reactions for the synthesis of aromatic or (partially) saturated heterocycles was proven in countless examples.^5^ Among the aromatic systems isoxazole, pyrrole, pyrazole, and triazole derivatives constitute the most important compound classes. In 1977 J. Rebek Jr. et al. smoothly prepared a “Münchnone” starting from l‐hydroxyproline and employed it for the synthesis of mitosene derivatives (Scheme 14).^72^

For asymmetric syntheses of heterocycles and their subsequent products the concerted process and the resulting stereospecificity are important elements of stereocontrol, thus enforcing the transfer of the configuration of dipolarophiles or 1,3‐dipoles (where possible) to the products. In addition, effective methods have been developed during the past decades to control diastereoselectivity and enantioselectivity. Chiral auxiliaries attached to dipolarophiles or 1,3‐dipoles were employed as well as enantioselective catalysis with metal complexes or organocatalysts. Numerous review articles testify to the tremendous progress made during the last 50 years.^73^

**Scheme 15:**
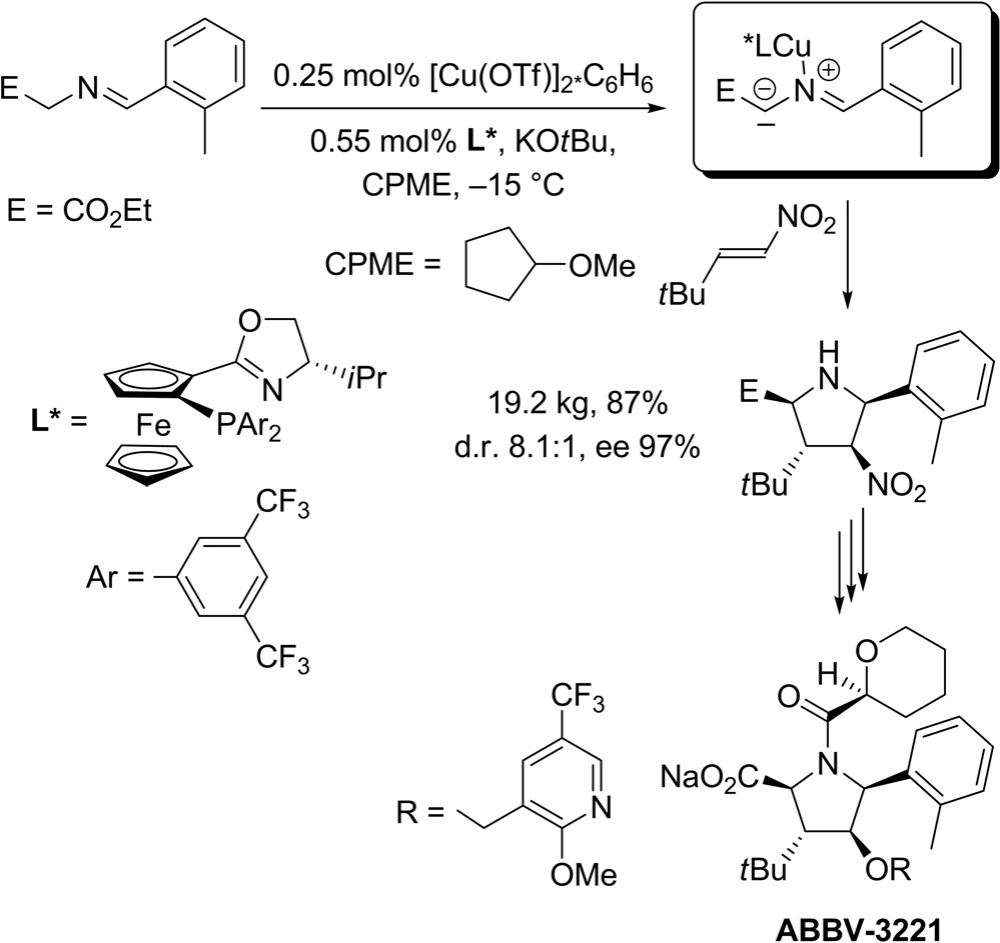
Catalytic enantioselective (3+2) cycloaddition of an in situ generated azomethine ylide with a nitroalkene performed on a multi‐kilogram scale.

The compound class of azomethine ylides—an unknown entity before the classification was established by R. Huisgen—has great impact on the synthesis of pyrrolidine derivatives, and these 1,3‐dipoles have been employed in many elegant applications for the preparation of natural products, drugs, and agrochemicals. A recent example depicted in Scheme 15 presents the preparation of a pyrrolidine building block on a multi‐kilogram scale.^74^

This transformation is characterized by the following features: in situ generation of the azomethine ylide by an easily available imine precursor; coordination of the required copper(I) triflate to an enantiomerically pure phosphane ligand; high efficacy of this catalytic system with respect to the amount of catalyst, diastereoselectivity (*exo/endo* ratio 8.1:1) and enantioselectivity (*ee* 97 %); high practicability. The experiment provided 19.2 kg of the desired stereoisomer (yield 87 %), which was converted to the drug candidate ABBV‐3221 in a few steps. This example is impressive in all aspects and benefits from the vast experience with asymmetric 1,3‐dipolar cycloadditions gained in the past decades.^73^

Besides azomethine ylides, carbonyl ylides,^75^ and thiocarbonyl ylides,^76^ mainly nitrones and nitrile oxides have been employed as 1,3‐dipoles to prepare isoxazole derivatives and to convert these into synthetically useful building blocks. G. Stork and J. E. McMurry very early used a nitrile oxide–alkyne cycloaddition to synthesize a specifically substituted isoxazole required as aldol‐equivalent building block for the synthesis of steroid derivatives.^77^ J. J. Tufariello applied nitrone–alkene cycloadditions for the synthesis of alkaloids,^78^ whereas R. V. Stevens et al.^79^ investigated nitrile oxide cycloadditions in their model studies towards vitamin B_12_. An intramolecular nitrile oxide cycloaddition was one of the key steps in the synthesis of racemic biotin reported by P. J. Confalone and co‐workers.^80^

**Scheme 16:**
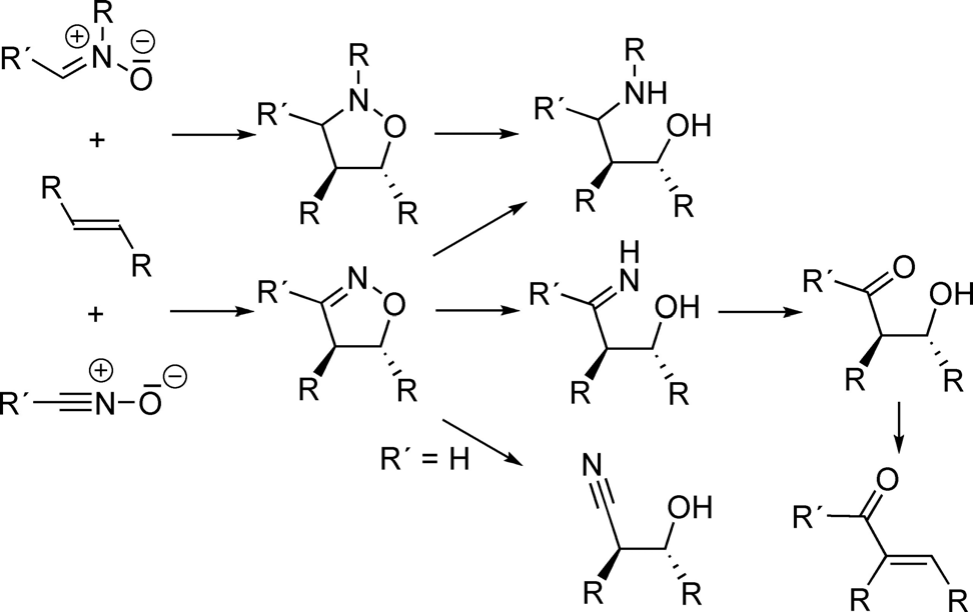
Syntheses and transformations of isoxazole derivatives into synthetically valuable acyclic products.

Systematic studies of the ring‐opening reactions of isoxazoline and isoxazolidine derivatives were mainly performed by the groups of V. Jäger^81^ and later by A. P. Kozikowski^82^ and D. P. Curran.^83^ By cleavage of the N−O bond, γ‐amino alcohols, β‐hydroxy ketones (aldol equivalents), α,β‐unsaturated carbonyl compounds, β‐hydroxy nitriles, and other intermediates can be prepared (Scheme 16). The stereoselectivities generated in the cycloaddition step and the subsequent reactions result in products with defined configurations. These synthetic methods were applied in numerous syntheses of natural products and their analogues, in particular for amino sugars, pyrrolizidine, and indolizidine alkaloids.^84^ Intramolecular versions of the (3+2) cycloaddition often led to increased stereoselectivity and enhanced efficiency of the reaction. For instance, the “detour” via isoxazolines to β‐hydroxy ketones was crucial for the synthesis of erythronolide A^85^ and other biologically active natural products.^86^ The meanwhile established protocols for catalytic enantioselective Huisgen reactions^73^ have strongly enhanced the significance of these synthetic methods.

## 6. Huisgen Reactions in Biosyntheses

**Scheme 17:**
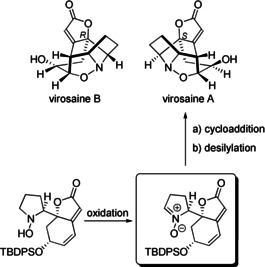
Virosaine A and virosaine B, two natural products with an isoxazolidine substructure and the key step of the synthesis of (−)‐virosaine A by K. Gademann et al.

The versatility of (3+2) cycloadditions in organic syntheses led to the question whether Nature also employs this reaction type in biosynthesis. In 2013, the two pseudo‐enantiomers virosaine A and B could in fact be isolated and characterized from branches and leaves of *Flueggea virosa* (Scheme 17). An intramolecular nitrone cycloaddition was proposed for the formation of the isoxazolidine ring.^87^ K. Gademann et al. succeeded in synthesizing virosaine A relying on this key step to connect the cyclic substructures under very mild conditions.^88^ Therefore, it is very likely that the (3+2) cycloaddition is also involved in the biosynthetic pathway.

**Scheme 18:**
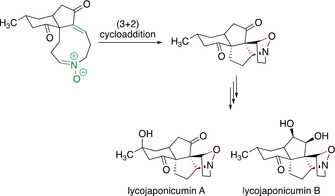
Potential intramolecular (3+2) cycloaddition as a key step in the biosynthesis of lycojaponicumin A and B.

These and other investigations suggest that Huisgen reactions could also be important for other biosynthetic pathways.^89^ In 2013 theoretical investigations by E. H. Krenske, A. Patel, and K. N. Houk^90^ indicated that also the biosynthesis of lycojaponicumin A and B could proceed via an intramolecular (3+2) cycloaddition of a nitrone (Scheme 18). Calculations at the M06‐2X/def2‐TZVPP//M06‐2X/6‐31+G(d,p) level show that the cycloaddition in question should be possible via hydrogen‐bond activation within the active site of an enzyme. A similar conclusion was drawn by the group of D. J. Tantillo: quantum‐mechanical calculations reveal that the (3+2) cycloaddition could also be of importance in the biosynthesis of alkaloids such as flueggine A.^91^

**Scheme 19:**
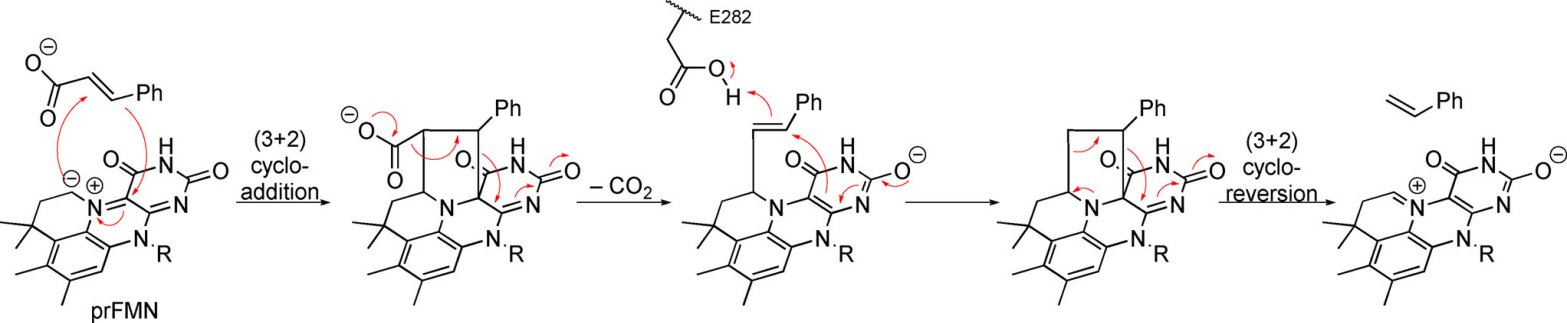
Proposed mechanism for the first enzymatic (3+2) cycloaddition and cycloreversion.

In 2015, D. Leys and colleagues finally identified the first biocatalyzed sequence of (3+2) cycloaddition and cycloreversion (Scheme 19):^92^ In this decarboxylation reaction, cinnamic acid derivatives are converted into styrene. Mechanistically, first a 1,3‐dipolar cycloaddition takes place between the cinnamic acid derivative and the cofactor prFMN (an azomethine ylide). After a Grob‐type fragmentation and a protonation by a glutamic acid residue, styrene is released in a (3+2) cycloreversion process.

In 2020, a group of researchers around Jiménez‐Barbero, A. L. Cortajarena, and F. P. Cossío were finally able to design a “Huisgenase” that is capable of catalyzing a (3+2) cycloaddition between azomethine ylides and nitroalkenes.^93^ In this enzymatic reaction between methyl *N*‐benzylidene glycinate and (*E*)‐nitrostyrene up to four racemic cycloadducts can be formed. The diastereoselectivity can be improved when slightly more rigid enzymes are employed.

## 7. The Click Chemistry Concept

The Huisgen reaction was already a classic method for the synthesis of heterocyclic compounds when this research area received a tremendous push from the concept of click chemistry. In 2001 K. B. Sharpless et al. described their conditions for an “ideal reaction=click reaction”, which should provide libraries of compounds as simply and efficiently as possible.^94^ The “handful of good reactions” should be characterized by broad applicability and ease of performance, they should not afford troublesome side‐products and should proceed close to completion. The Sharpless review already lists the 1,3‐dipolar cycloaddition and the Diels–Alder reaction as good candidates. In this context it is remarkable to recall a sentence in R. Huisgen's autobiography published a few years earlier: “*The dream of reactions proceeding quantitatively under mild conditions without the need of catalysis is often fulfilled by concerted cycloadditions*”.^95^

**Figure 5:**
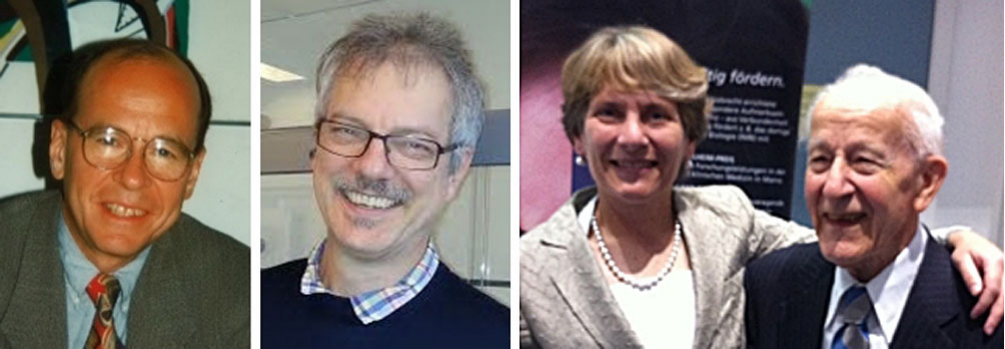
K. Barry Sharpless (1995), Morten Meldal (2016), and Carolyn R. Bertozzi with Rolf Huisgen (2012).

**Scheme 20:**
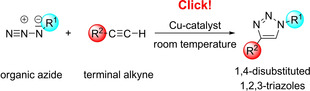
Copper‐catalyzed click reaction of organic azides with terminal alkynes to give 1,4‐disubstituted 1,2,3‐triazoles.

Ironically, it was a *catalyzed* reaction that initiated the breakthrough of the click chemistry concept. In 2002 the groups of M. Meldal^96^ as well as of V. V. Fokin and K. B. Sharpless^97^ (Figure 5) published their first reports on copper‐catalyzed azide–alkyne cycloadditions which afterwards became so popular and enjoyed a success story in chemistry, life science, and materials science. The mild reaction conditions, the high yields, and the formation of only one regioisomer was decisive for this great success. Whereas the thermal azide–alkyne cycloadditions generally furnish mixtures of regioisomers,^98^ the copper‐catalyzed reactions provide only the 1,4‐disubstituted 1,2,3‐triazole derivatives (Scheme 20). This is due to the complex mechanism which does not actually involve a concerted (3+2) cycloaddition but follows a stepwise process with several organocopper intermediates.^99^ The importance of the azide–alkyne cycloadditions is also supported by the ready availability of the two required components, their general inertness to other functional groups, and the mild reaction conditions, which even allow transformations at room temperature in aqueous (biological) systems. This type of click reaction allows the (frequently quantitative) connection of two different, often quite complex molecules, for instance of proteins with fluorescence markers, of azido‐substituted carbohydrate derivatives with cell surfaces, and of building blocks leading to new polymers.^100^ For this reason the copper catalysts, ligands, solvents, and reaction conditions were adjusted to the corresponding application, resulting in many variants of the applied procedures. The newly gained importance of organic azides also initiated a renaissance of this compound class.^101^

**Scheme 21:**
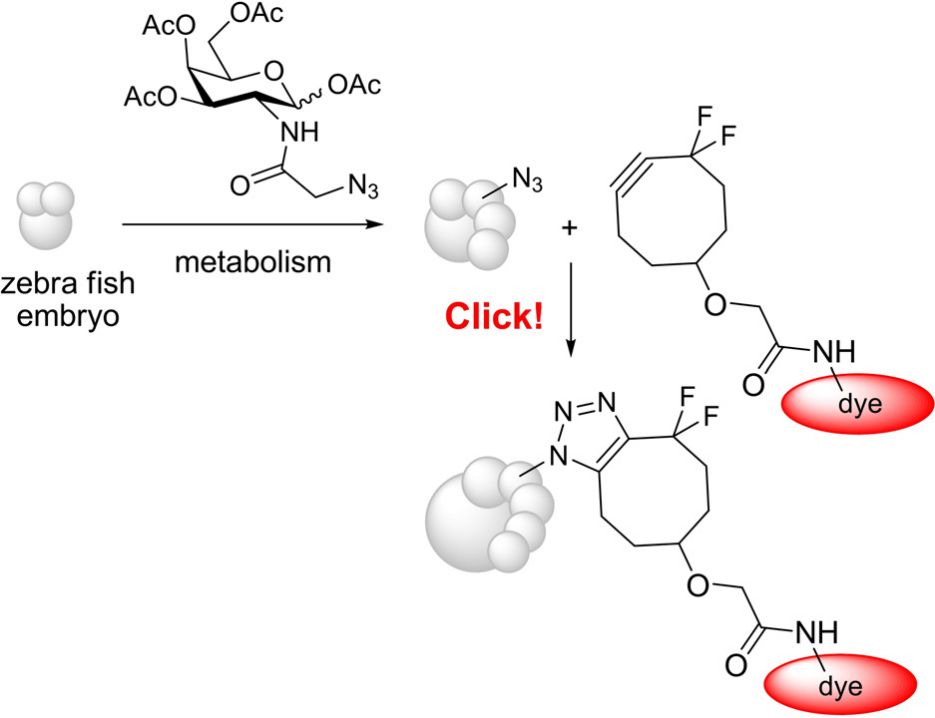
Incorporation of an azido‐substituted carbohydrate into a developing zebrafish embryo (schematic illustration) and Huisgen reaction with a cyclooctyne derivative bearing a fluorescence dye.

In 2004 during her search for bioorthogonal reactivities C. R. Bertozzi (Figure 5) found that cyclooctyne derivatives undergo a copper‐free Huisgen reaction with azides.^102^ It should be recalled that the observed high reactivity of aryl azides to strained dipolarophiles^103^ led to the development of the concept of 1,3‐dipolar cycloadditions (see above).^24^ For these azide–cyclooctyne cycloadditions many variations were devised,^104^ and their biocompatibility was impressively proven in many applications. As an example, C. R. Bertozzi et al. incorporated azido‐substituted galactose amine derivatives into living zebrafish embryos and marked them with a cyclooctynyl‐substituted fluorescence dye (Scheme 21), thus enabling in vivo imaging during the development of the embryo.^105^ The two fluoro substituents were introduced to enhance the reactivity of the cyclooctyne.

The overwhelming success of the azide–alkyne cycloadditions in the life sciences initiated a search for other suitable 1,3‐dipoles with complementary reactivities. Among the examined compound classes were nitrile imines, nitrile oxides, “Sydnones”, “Münchnones”, and nitrones, but none of them gained the broad applicability of azides. Recently, R. T. Raines et al. investigated diazo carbonyl compounds which show an interesting reactivity profile since they are less sensitive to steric effects.^106^ This completes the cycle because E. Buchner used diazo carbonyl compounds to perform the first 1,3‐dipolar cycloadditions (see Scheme 3) and diazoalkane reactions were the trigger to guide the group of R. Huisgen finally to the concept of 1,3‐dipolar cycloadditions.

## 8. Conclusions

During the last 60 years the Huisgen reaction, also known as the 1,3‐dipolar cycloaddition, has become an indispensable tool of chemistry and many colleagues think that it should have been honored by the Nobel prize. After introducing the general concept, most of the synthetic and mechanistic investigations were first performed in the Munich laboratories under the guidance of Rolf Huisgen, but shortly thereafter the 1,3‐dipolar cycloaddition started its triumphal procession around the world. In particular, azomethine ylides, azomethine imines, nitrile ylides, and nitrile imines, unknown 1,3‐dipoles before 1960, as well as nitrile oxides and the mesoionic “Sydnones” and “Münchnones” have evolved into versatile and frequently used building blocks used by synthetic chemists in (3+2) cycloadditions.

The Huisgen reaction was also frequently investigated by theoretical chemists, who examined it from different points of view—also in order to examine the validity of their methods. The analogy to the electronically equivalent Diels–Alder reactions was evident. The concerted reaction mechanism already suggested in 1960 was regularly confirmed by experiment and theory and it explains the outcome of almost all (3+2) cycloadditions.

The Huisgen reaction is employed for the preparation of heterocycles and is also involved in many applications for the synthesis of natural products and biologically active compounds. Today we know that Nature also uses the Huisgen reaction. The corresponding biosynthetic pathways have been proposed, supported by computational studies, and in part imitated in total syntheses. In 2001, K. B. Sharpless suggested the concept of click chemistry, but without (3+2) cycloadditions, in particular without the discovery of the catalyzed azide–alkyne cycloadditions, this would have been a meager proposal. Thanks to the brilliant ability of Rolf Huisgen^107^ to systematically order previously unrelated results, to go into great detail, and to expand the new knowledge in a creative manner, this important branch of chemistry developed the way it did.

*“To unveil the truth is not conceivable without divergence of opinions, since the truth cannot be recognized, in its full extent, in one step, and by all at the same time. Each step with which the natural scientist is seemingly approaching his goal leads him to the entrance of a new labyrinth” –* Alexander von Humboldt, Berlin (1828)^108^ (translated by the authors).

## Conflict of interest

The authors declare no conflict of interest.

## Biographical Information

*Martin Breugst (right) studied chemistry at the Ludwig‐Maximilians‐Universität in Munich (Germany). He completed his PhD there in 2010 under the guidance of Prof. Dr. Herbert Mayr in the area of physical‐organic chemistry. Subsequently, he worked as a Feodor‐Lynen postdoctoral fellow of the Alexander‐von‐Humboldt foundation with Prof. Dr. Kendall N. Houk at the University of California, Los Angeles (UCLA) on different aspects of computational organic chemistry. In 2013, he started his independent career as a Liebig fellow of the Fonds der Chemischen Industrie at the Department of Chemistry of the University of Cologne. After his habilitation in 2017, he served as an interim professor at the University Regensburg and RWTH Aachen University. His research is currently focused on noncovalent interactions and experimental and computational elucidation of reaction mechanisms. Hans‐Ulrich Reissig studied chemistry at the Ludwig‐Maximilians‐Universität in Munich. He worked on diazoalkane cycloadditions under the guidance of Prof. Dr. Rolf Huisgen and was promoted to Dr. rer. nat. in 1978. After a postdoctoral stint with Prof. Dr. Edward Piers at the University of British Columbia, Vancouver (Canada), he started independent research for his habilitation at the Universität Würzburg (mentor: Prof. Dr. Siegfried Hünig). He has held professorships in Darmstadt (from 1986), in Dresden (from 1993) and in Berlin (1999–2015). His research activities concentrated on the development of stereoselective synthetic methods (donor–acceptor cyclopropanes, samarium diiodide induced cyclizations), heterocyclic chemistry (alkoxyallenes), and natural product synthesis (rubromycin, strychnine). He was elected as a corresponding member of the Bavarian Academy of Sciences and Humanities, received the Liebig Memorial Medal of the GDCh, and has been honorary member of the Polish Chemical Society since 2017*.